# 抗肺癌药物相关间质性肺病的研究进展

**DOI:** 10.3779/j.issn.1009-3419.2025.106.11

**Published:** 2025-04-20

**Authors:** Zhimin XIAO, Yan GU

**Affiliations:** 010107 呼和浩特，内蒙古医科大学附属医院呼吸与危重症医学科; Department of Respiratory and Critical Care Medicine, The Affiliated Hospital of Inner Mongolia Medical University, Hohhot 010107, China

**Keywords:** 肺肿瘤, 抗肺癌药物, 药物性间质性肺病, 酪氨酸激酶抑制剂, 免疫检查点抑制剂, 多学科协作, Lung neoplasms, Anti-lung cancer drugs, Drug-induced interstitial lung disease, Tyrosine kinase inhibitors, Immune checkpoint inhibitors, Multidisciplinary collaboration

## Abstract

全球范围内肺癌的发病率和病死率均为最高。随着医学研究的进展及治疗手段的丰富，患者的生存期得以改善，但仍需关注用药的相关不良反应，其中药物性间质性肺病（drug-induced interstitial lung disease, DI-ILD）最为常见，抗肺癌药物相关ILD是指经过抗肺癌治疗出现肺间质炎症和纤维化的病变，而不良预后以及较高的病死率与此有关；并且抗肺癌药物相关ILD的临床管理也需要多学科协作（multi-disciplinary treatment, MDT）模式应对其复杂的诊断和治疗。本文综述了抗肺癌药物相关ILD在流行病学、分子生物学、基因组学和遗传学、诊断以及治疗等方面的研究，为临床医生提供参考，旨在尽早发现抗肺癌药物相关ILD，改善患者预后和生活质量。

## Abstract (en)

Lung cancer is the cancer with the highest incidence and mortality rate worldwide. In addition to the diversified treatment and prolonged lifespan in view of the development of medical technology, the side effect of medicine should not be ignored. Drug-induced interstitial lung disease (DI-ILD) is also commonly encountered during this process, and ILD triggered by the treatment of lung cancer characterized by the inflammation and scarring of lung tissue after the antitumor treatment in lung cancer leads to a poor prognosis and high mortality. The diagnosis and treatment of ILD caused by anti-lung cancer agents remains challenging in clinical settings and requires joint efforts from multidisciplinary team (MDT). This review systematically updates the epidemiology, molecular pathogenesis, genomics/genetics study, diagnosis and treatment of ILD related to anti-lung cancer agents. By the integration of the latest evidences, the paper offers clinical work references for early diagnosis of ILD related to anti-lung cancer agents to enhance the survival and quality of life of the lung cancer patients.

肺癌是全球发病率和死亡率较高的恶性肿瘤之一^[1,2]^，治疗包括手术治疗、化疗、放疗、靶向治疗及免疫治疗等。抗肺癌药物的应用为治疗提供了一定的方案。然而，部分药物可以引起药物性间质性肺病（drug-induced interstitial lung disease, DI-ILD），随着靶向药物、免疫治疗和抗体偶联药物（antibody-drug conjugate, ADC）的临床推广，ILD作为一种潜在副作用逐步进入人们的视野^[3]^。抗肺癌药物延长了肺癌患者的生存期，但是一些抗肺癌药物具有致ILD的药物不良反应，致死率高，导致治疗相关死亡率增加，已经成为临床医师不可忽视的问题。鉴于抗肺癌药物致ILD的严重程度，本文拟对涉及抗肺癌药物致ILD方面进行回顾和综述，以便为今后研究及临床提供一定的指导意义。

## 1 流行病学

不同治疗方案所致I LD的发生率为1%-60%，其中抗肿瘤药物在DI-ILD致病因素中的构成比达23%-51%，抗肺癌药物相关ILD发生率约为2.52%^[3-5]^。不同类别的抗肺癌药物引发ILD的发生率存在差异，其中，化疗药物、酪氨酸激酶抑制剂（tyrosine kinase inhibitors, TKIs）以及免疫检查点抑制剂（immune checkpoint inhibitors, ICIs）是常见的药物类型。化疗药物如铂类（顺铂、卡铂、奥沙利铂）、紫杉类（紫杉醇、多西紫杉醇）、抗代谢类（吉西他滨、培美曲塞）、抗生素类（博来霉素）；TKIs类主要有表皮生长因子受体（epidermal growth factor receptor, EGFR）-TKIs一代（吉非替尼、厄洛替尼）、EGFR-TKIs二代（阿法替尼、达克替尼）、EGFR-TKIs三代（奥希替尼、阿美替尼、伏美替尼），间变性淋巴瘤激酶（anaplastic lymphoma kinase, ALK）-TKIs一代（克唑替尼）、ALK-TKIs二代（塞瑞替尼、布格替尼、阿来替尼、恩沙替尼）、ALK-TKIs三代（洛拉替尼），ROS原癌基因1（ROS proto-oncogene 1, ROS1）- TKIs一代（克唑替尼、恩曲替尼），ROS1-TKIs二代（瑞波替尼）、ROS1-TKIs三代（他雷替尼）；ICIs类主要有程序性死亡受体1（programmed cell death protein 1, PD-1）（信迪利单抗、替雷丽珠单抗、卡瑞利珠单抗、纳武利尤单抗、帕博利珠单抗），程序性死亡配体1（programmed cell death ligand 1, PD-L1）（贝莫苏拜单抗、阿替利珠单抗），ADC类药物主要有人表皮生长因子受体2（human epidermal growth factor receptor 2, HER_2_）-ADC（恩美曲妥珠单抗、德曲妥珠单抗）、滋养层细胞表面抗原（trophoblast cell surface antigen 2, TROP_2_）-ADC（芦康沙妥珠单抗），均可出现ILD。各抗癌药物诱发ILD的发生率有差异（表1^[6-33]^）。这些结果突显了在抗肺癌药物治疗下需关注监测和治疗DI-ILD的风险。

**表1:** 不同类别抗肺癌药物所致间质性肺病的发生率

Category of anti-lung cancer drugs	Incidence of ILD (%)
Chemotherapy agents	
Platinum-based agents^[6,7]^	1.5-10.0
Taxanes^[7,8]^	0-8.3
Antimetabolites^[3,8]^	1.1-22.6
Antibiotics^[3,9]^	6.8-21.0
TKIs	
EGFR-TKIs	
First-generation^[3,10,11]^	0-5.9
Second-generation^[11,12]^	0.4-4.7
Third-generation^[11,13-16]^	0.4-12.3
ALK-TKIs	
First-generation^[17,18]^	1.2-3.7
Second-generation^[18-20]^	0.08-4
Third-generation^[18]^	0-1
ROS1-TKIs	
First-generation^[17,18,21]^	0-3.7
Second-generation^[22]^	0-1
Third-generation^[23]^	0-1.2
ICIs	
PD-1^[3,14,24-29]^	1.4-18.8
PD-L1^[3,30,31]^	1.1-4.5
ADCs	
HER_2_-ADC^[32,33]^	0.5-28
TROP_2_-ADC^[32]^	3.4

Geographical bias exists: Japan reports a higher incidence of DI-ILD caused by anti-lung cancer drugs compared to Western countries, possibly due to HLA gene polymorphisms or differences in diagnostic standards. Data are derived from multicenter retrospective studies and clinical trials. DI-ILD: drug-induced interstitial lung disease; TKIs: tyrosine kinase inhibitors; EGFR: epidermal growth factor receptor; ALK: anaplastic lymphoma kinase; ROS1: ROS proto-oncogene 1; PD-1: programmed cell death protein 1; PD-L1: programmed cell death-ligand 1; HER_2_-ADC: human epidermal growth factor receptor 2 antibody-drug conjugate; TROP_2_-ADC: trophoblast cell surface antigen 2 antibody-drug conjugate; ICIs: immune checkpoint inhibitors.

## 2 分子机制

### 2.1 药物与肺细胞之间的相互作用

铂类药物通过与DNA结合，干扰其复制与转录，激活p53信号通路，诱导细胞周期停滞与凋亡以抑制肿瘤细胞增殖^[34,35]^。靶向药物（EGFR-TKIs、ALK-TKIs）通过抑制特定的信号通路，如EGFR和ALK的信号通路^[36,37]^，来阻断肿瘤细胞的增殖和生存信号，从而抑制肿瘤的生长和扩散。ICIs类药物通过阻断免疫细胞上的抑制性受体（如PD-1）与主要在肿瘤细胞上表达的配体（如PD-L1）之间的相互作用，引起的抑制信号来激活T细胞以对抗肿瘤^[38]^。

抗肺癌药物诱导肺间质纤维化的细胞-分子机制尚未明确，目前研究^[39,40]^表明可能与以下机制有关：（1）线粒体功能障碍介导的氧化应激与炎症反应：线粒体功能障碍是抗肺癌药物（如铂类、TKIs）诱导肺损伤的核心机制之一。药物可能引起肺泡上皮细胞线粒体DNA损伤，进而通过氧化磷酸化异常而导致活性氧（reactive oxygen species, ROS）过量释放。例如，吉非替尼可上调线粒体ROS生成，激活NLRP3炎性小体，并释放DAMPs，诱导肺泡巨噬细胞和中性粒细胞的募集，最终诱发肺部炎症。高浓度的ROS可激活NLRP3炎性体，促分泌白细胞介素（interleukin, IL）-1β、IL-6和抑制肿瘤坏死因子-α（tumor necrosis factor alpha, TNF-α）等促炎性因子，加重肺部炎症以及肺纤维化；（2）上皮充间质转化（epithelial-mesenchymal transition, EMT）：EMT是一种细胞转化过程，使上皮细胞失去其原来的一些特性而表现间质细胞的特性，这种转化引起细胞形态和细胞间质的重构，导致肺组织发生纤维化，抗肺癌药物诱导的EMT可能促使肺间质纤维化；（3）细胞凋亡：抗肺癌药物可能引起肺组织细胞凋亡进而发生肺纤维化；（4）细胞外基质（extracellular matrix, ECM）重塑：抗肺癌药物可能引起肺组织内EMC合成和降解异常导致ECM异常蓄积而引发纤维化；（5）巨噬细胞极化与辅助性T细胞2型（T-helper 2 cells, Th2）细胞因子作用：化疗药物可将肺部组织巨噬细胞转变成M2型，M2型肺泡巨噬细胞通过转化生长因子-β（transforming growth factor-beta, TGF-β）、IL-4、IL-10和IL-13等Th2细胞因子的释放，介导成纤维细胞向肌成纤维细胞转化，使ECM（胶原蛋白和纤连蛋白）过度产生，其中TGF-β可以直接刺激EMT过程，还可通过Smad2/3通路上调NOX4以促进氧化应激产生以及纤维化加重。

### 2.2 药物对肺纤维化过程的影响

肺纤维化的发生与抗肺癌药物对关键信号通路的调控密切相关。在病理生理过程中，IL-4、IL-10、IL-13和TGF-β等主要的细胞信号因子起重要的上游控制作用^[39]^。IL-4、IL-10和IL-13主要通过激活ECM途径来增加胶原、纤维蛋白和纤连蛋白等ECM组成成分的生物合成，从而使肺结构重建并产生纤维化。TGF-β是纤维化级联反应的中心调控因子，不仅可通过上调ECM成分的生物合成间接参与肺组织纤维化过程，还可通过调节免疫细胞表型转化为Th2淋巴细胞及M2巨噬细胞的分化 。Th2细胞分泌的IL-4、IL-13通过级联转导。增强M2型巨噬细胞表达，并导致TGF-β和血小板源性生长因子（platelet-derived growth factor, PDGF）的释放。PDGF是一种具有极大增殖功能的促增殖因子，它能够更高效地增强成纤维细胞活性和异常增殖。这个基于TGF-β、IL-4和IL-13的肺纤维化正反馈机制持续加重纤维化过程^[39]^。TGF-β、IL-4和IL-13也可以上调促氧化酶如烟酰胺腺嘌呤二核苷酸磷酸氧化酶（nicotinamide adenine dinucleotide phosphate oxidase, NOX）和环氧化酶-2（cyclooxygenase-2, COX-2），增加ROS的产生，从而加重氧化应激反应和纤维化过程（图1）。

**图1:**
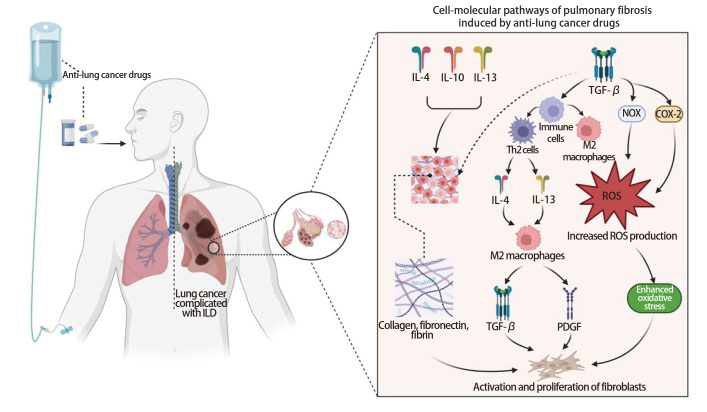
抗肺癌药物诱导肺纤维化的细胞-分子通路图

细胞培养实验^[41]^提示，细胞经顺铂处理后，TGF-β 表达显著增加，同时伴随ECM成分增加。动物实验^[42,43]^表明，博来霉素诱导的小鼠肺纤维化，模型显示肺组织 TGF-β表达增加，肺成纤维细胞增殖并转分化为肌成纤维细胞，致大量ECM成分聚集，使肺组织出现显著的纤维化病理变化。

## 3 基因组学和遗传学研究

### 3.1 遗传多态性介导的药物代谢差异与肺损伤风险

DI-ILD相关的抗肺癌药物遗传多态性与药代动力学及不良反应相关的文献报道较多。细胞色素P450（cytochrome P450, CYP450）是一种亚铁血红素蛋白家族成员，它们的基因有着极高的遗传变异性，在抗肺癌药物代谢中起着重要作用^[44]^。个体对一种药物的代谢能力可因CYP450的基因遗传多态性存在异质性，进而导致药物效应呈现个体化差异。研究^[45]^发现，与肺纤维化易感相关的CYP2D6、CYP2C9、CYP2C19等基因型的特定多态性改变明显增加。同时，日本人群遗传流行病学研究^[46]^发现，携带HLA-DRB1*04:05等位基因的化疗患者发生DI-ILD的风险增加（OR=1.74, 95%CI: 1.30-2.32），其可能的病理机制为HLA介导的异常抗原呈递而导致适应性免疫紊乱。鉴于该等位基因在东亚人群中的高频率出现的遗传特征，其可构成该地区人群DI-ILD高发病率的重要分子遗传学基础。

### 3.2 全基因组关联研究（genome-wide association studies, GWAS）的突破性进展

GWAS通过系统性筛查遗传标记，发现了与肺纤维化风险密切相关的单核苷酸多态性（single nucleotide polymorphism, SNP）^[47]^。近年来，GWAS不断探索DI-ILD发病机制的进展^[48]^，尤其是MUCSB启动子区rs35705950多态性（OR=5.06）已被GWAS认为是肺纤维化发生的最高度易感多态性，其通过MUCSB的异常高表达所致的内质网应激、肺泡上皮损伤，可能加剧与环境接触或者药物诱发的毒性损伤；另外，还发现GWAS的中度效应风险位点，如TOLLIP的rs171521887（OR=1.48），可通过调控TGF-β信号通路以及通过Toll样受体（Toll-like receptor, TLR）介导的固有免疫反应而参与致病过程，该研究提示其作用可能影响自噬异常。这些研究为基于遗传的个体化治疗提供了新的可能。

## 4 诊断方法

### 4.1 临床检查

抗肺癌药物引起的DI-ILD症状多样且非特异，出现时间跨度大（数日-数年），常见症状如咳嗽、劳力性呼吸困难、发热^[49,50]^。与肺癌症状相互影响，症状隐匿而容易被忽视，其中劳力性呼吸困难为关键监测指标，恶化提示病情进展。监测呼吸频率、SpO_2_的变化以及乏力、胸痛、胸闷，同时注意胸部听诊可能闻及的湿啰音或Velcro啰音改变^[4,49]^。上述症状和体征的出现通常被认为是肺部间质发生了药物不良反应的预警。

### 4.2 辅助检查

尽管抗肺癌药物所致ILD在影像学、实验室检测和组织病理学表现各异且非特异性，胸部高分辨率CT（high-resolution computed tomography, HRCT）仍是核心诊断工具，可精准捕捉肺间质纤维化与炎症变化征象。

对于诊断ILD来说，人工智能（artificial intelligence, AI）影像评估改变了现有模式。利用目前最具有优势的深度学习算法即卷积神经网络（convolutional neural network, CNN）可以准确判断出特定的病理改变（纤维化病灶的位置和炎性浸润的区域量化），并由此建议下一步临床决策。值得提及的是，Fibresolve系统即根据影像学特点（CT影像上纤维化的纹理及分布特征）来评估患者的纤维化程度，在无组织病理支持的情况下，该系统可以将特发性肺纤维化（idiopathic pulmonary fibrosis, IPF）的敏感性和特异性提升，利用影像组学中的多维特征对不同CT影像的比较分析已在非典型病例中作为类似组织学判读，具有一定的临床评估价值^[51,52]^。例如，一项纳入3018例患者的多中心回顾性研究^[53]^开发的AI算法（ScreenDx-LungFibrosis™），通过识别肺纤维化特征（如网状结构、牵拉性支气管扩张），在独立队列分析中发现诊断肺纤维化的敏感性为91.3%（95%CI: 89.0%-93.3%），特异性为95.1%（95%CI: 94.2%-96.0%），受试者工作特征（receiver operating characteristic, ROC）曲线下面积（area under the curve, AUC）达0.997，且平均处理时间只需27.6 s，结果优于单独的影像学评估方法（P<0.001），提示该算法可帮助临床医师评价DI-ILD的初步筛查。

上述进展预示着ILD诊疗模式正向着智能方向发展，依靠AI影像组学技术，让诊断方式由经验模式逐步过渡为多指标量化的形式，优化了临床决策的效率；金标准组织病理学检查确定病变性质与严重程度。而HRCT影像与病理学检查结合仍然是目前对ILD进行早期诊断及疾病严重程度评估的首选方法。常见影像学、病理学表现如表2^[4,49,54-62]^。

**表2:** 抗肺癌药物相关ILD常见影像学与病理学表现

Pattern	HRCT findings	Histopathological features
DAD^[4,49,54-57]^	Exudative phase: bilateral diffuse GGO with consolidation; Proliferative/fibrotic phase: reticular opacities, traction bronchiectasis, volume loss	Exudative phase: hyaline membrane formation, alveolar edema; Proliferative/fibrotic phase: fibroblast infiltration, alveolar collapse, honeycombing
Simple pulmonary eosinophilia^[49,54,55]^	Transient, migratory non-segmental consolidation or GGO	Massive eosinophilic infiltration in alveoli and interstitium, may accompany hyaline membranes
OP^[4,49,54-58]^	Patchy consolidation/GGO, peribronchovascular or peripheral distribution, "reverse halo sign"	Intra-alveolar masson bodies (fibroblastic/myofibroblastic granulation tissue), mild-moderate lymphocytic infiltration
HP^[4,49,54-55,58]^	Diffuse GGO, centrilobular micronodules, mosaic attenuation	Peribronchiolar granulomas, chronic interstitial inflammation and fibrosis
NSIP^[4,49,54-60]^	Bilateral GGO predominantly in the middle and lower lungs, may progress to reticular opacities, traction bronchiectasis	Uniform interstitial inflammation with type II alveolar epithelial hyperplasia, without structural destruction, and interstitial lymphocyte/plasma cell infiltration
UIP^[55,57-58,61,62]^	Subpleural honeycombing, reticular opacities, traction bronchiectasis	Patchy fibrosis with architectural distortion (honeycombing), fibroblast foci distributed at the edges of fibrosis

DAD: diffuse alveolar damage; OP: organizing pneumonia; HP: hypersensitivity pneumonitis; NSIP: nonspecific interstitial pneumonia; UIP: usual interstitial pneumonia; HRCT: high-resolution computed tomography; GGO: ground-glass opacity.

### 4.3 其他检查

实验室检查、动脉血气测定、肺功能测定、支气管镜检查下可进行支气管肺泡灌洗液（bronchoalveolar lavage fluid, BALF）、支气管镜透壁肺活检（transbronchial lung biopsy, TBLB）、支气管肺冷冻活检（transbronchial lung cryobiopsy, TBLC）等方法对于抗肺癌药物相关DI-ILD的诊断与鉴别诊断具有很好的辅助作用，并可以全面评估患者的病情状态（表3^[4,49,50,55]^）。近年来，因创伤性少、安全性较高（不良事件发生率低和住院周期短）、相对于外科肺活检（surgical lung biopsy, LB）对ILD的病理诊断价值不比后者差等优点，TBLC已作为ILD诊断的替代或补充方案逐渐开展及应用^[63]^。在经验丰富的呼吸介入团队操作下，TBLC经多学科协作（multi-disciplinary treatment, MDT）后的综合诊断率可超过80%以上^[64]^。这些检查手段共同形成了一整套检测系统，协助临床医生更加精准地掌握病情，制定合理治疗方案。

**表3:** 其他检查的辅助信息表

Test	Brief description
Laboratory tests^[4,50,55]^	CBC, liver/kidney function, ESR, CRP, LDH, KL-6, PCT should be measured in cases of shock. Elevated KL-6 indicates alveolar epithelial injury (e.g. DAD).
Blood gas analysis^[55]^	Assess oxygenation and acid-base balance (pH, PaO_2_, PaCO_2_, HCO_3_^-^, BE), monitor risk of respiratory failure.
Pulmonary function tests^[4,55]^	Restrictive ventilatory impairment (TLC、FVC↓). DLCO suggests interstitial pathology. Contraindicated in respiratory failure.
BALF^[4,49]^	Lymphocytic alveolitis, predominantly CD8^+^ lymphocytes.
Bronchoscopy and biopsy^[4,49-50]^	TBLB or TBLC to obtain lung tissue, exclude infection or malignancy. Combined with HRCT and MDT to improve diagnostic accuracy.

CBC: complete blood count; ESR: erythrocyte sedimentation rate; CRP: C-reactive protein; LDH: lactate dehydrogenase; KL-6: Krebs von den Lungen-6; PCT: procalcitonin; PaO_2_: arterial oxygen partial pressure; PaCO_2_: arterial carbon dioxide partial pressure; HCO_3_^-^: bicarbonate ion concentration; BE: base excess; TLC: total lung capacity; FVC: forced vital capacity; DLCO: diffusing capacity of the lungs for carbon monoxide; CD8: cluster of differentiation 8; TBLB: transbronchial lung biopsy; TBLC: transbronchial lung cryobiopsy; MDT: multi-disciplinary treatment; BALF: bronchoalveolar lavage fluid.

### 4.4 新兴诊断技术的进展

随着个体化治疗技术改革的驱动，与抗肺癌药物有关的DI-ILD的诊断方法将逐步向多维度、多角度诊断模式进行优化。据报道^[65]^，机器整合生物信息的高效判断和预测模型（如综合分析使用朴素贝叶斯算法的多维血液标志物分析软件）对ILD进行判断和预测的能力明显高于传统的判断模型。中性粒细胞与淋巴细胞之比（neutrophil-to-lymphocyte ratio, NLR）是一种重要的炎症反应和纤维化前转换调节因子，这种平衡机制对于肺癌与ILD病理性变化的鉴别诊断表现出高效能（敏感性和特异性分别为99.9%和64.9%，AUC为0.833）。在多维度支持的判断中加入单核细胞与淋巴细胞之比（monocyte-to-lymphocyte ratio, MLR）（AUC为0.796）和血小板与淋巴细胞之比（platelet-to-lymphocyte ratio, PLR）（AUC为0.699）具有一定的判断价值。目前，NLR、MLR、PLR虽然在IPF与肺癌中肺腺癌纤维化中的效能也已被证明^[65]^，但在抗肺癌药物相关ILD中的判别效能尚未见文献报道，亟待研究。

## 5 治疗与展望

### 5.1 个性化治疗

抗肺癌药物致DI-ILD的严重程度可呈现从轻症到重症的异质性表现，其临床转归差异显著。参考美国国立癌症研究所通用不良事件术语第5.0版（National Cancer Institute-common terminology criteria adverse events 5.0, NCI-CTCAE v5.0）标准，DI-ILD分为5级：G1级（无症状，仅影像学异常）、G2级（有症状，轻度呼吸道症状）、G3级（需吸氧的重度症状）、G4级（危及生命）和G5级（死亡）。DI-ILD的治疗方案需分层管理，核心目标为控制炎症并改善肺功能^[4,55]^：（1）G1级：TKIs和ICIs可继续使用，但需密切监测（每2-3 d临床评估，每2-3周HRCT/肺功能）；ADC类药物（如T-DXd）需暂停至ILD缓解。若28 d内缓解可恢复原剂量，超过28 d则减量，49 d未缓解则永久停药。（2）G2级：立即停药，口服泼尼松1-2 mg/(kg·d)，3-5 d内改善则4-6周内减量；无改善则升级至G3级方案。ADC相关ILD需泼尼松龙≥1 mg/(kg·d)持续14 d，缓解后4-6周减量。（3）≥G3级：需氧疗或机械通气，甲泼尼龙1-2 mg/(kg·d)持续至症状缓解，8-12周内减停；严重病例可予甲泼尼龙500-1000 mg/d冲击3 d，后序贯泼尼松≥1 mg/(kg·d)≥2周。48-72 h无效时，联合英夫利昔单抗或免疫球蛋白。

针对不同治疗方案的选择，为临床决策提供依据：（1）糖皮质激素的优势是可以快速抑制炎症反应，改善急性期症状，尤其在机化性肺炎（organizing pneumonia, OP）中缓解率>70%，但它的局限是长期使用易诱发感染、骨质疏松；对纤维化进展抑制作用有限，非特异性间质性肺炎（nonspecific interstitial pneumonia, NSIP）缓解率为45.8%，过敏性肺炎（hypersensitivity pneumonitis, HP）缓解率为36.4%^[3]^。（2）在生物制剂治疗领域，英夫利昔单抗（Infliximab）的临床应用为激素耐药性肺间质病变提供了新型干预策略，其作用机制可能与抑制TNF-α介导的炎症反应有关，可使经糖皮质激素规范治疗仍病情持续进展的难治性病例的氧合指数得到显著改善^[66]^。但该疗法的风险谱系需审慎评估，循证证据多源于病例报告，缺乏高级别临床研究支持，可能诱发感染或罕见情况下加重ILD的情况^[67]^，并且高昂的治疗成本限制其广泛使用。

### 5.2 治疗优化

钟南山院士团队推动了肿瘤和呼吸病学的交叉学科研究^[68]^，带来肿瘤呼吸病学新理念，为探索新治疗理念创新DI-ILD治疗方法提供新方法。关于抗肺癌药物相关DI-ILD的治疗理念的创新，已从单纯糖皮质激素治疗转向精准化及多靶点联合治疗，一方面是抗纤维化药物的新突破，吡非尼酮和尼达尼布在IPF中疗效已得到肯定，适应证逐步拓宽至DI-ILD。回顾性研究^[69]^显示，围手术期吡非尼酮可降低肺癌合并IPF患者术后间质性肺疾病急性加重（acute exacerbation of ILD, AE-ILD）的发生风险，将尼达尼布联合化疗药物（卡铂和白蛋白紫杉醇）可以显著延缓肺癌合并IPF患者发生AE-IPF的时间间隔；通过数据和机器学习构建风险评估模型，预测发生DI-ILD的危险概率，实现治疗前充分告知和准备。另外一方面是结合基因变异、蛋白表达谱生物标志物等相关组学技术应用，预测基因变异、表达谱，基于影像组学及AI辅助CT影像分析^[70]^，实现对DI-ILD的早期识别与干预，提高诊断率与效率。

在药物控制与保护性方面，除对原有糖皮质激素治疗的不断优化外，也要不断探索新型的治疗方案，比如免疫抑制剂及生物制剂等；此外，特别需要注意药物之间的相互作用及安全性，以便给肺癌合并ILD患者提供更精确的个体化治疗。

### 5.3 MDT的深化

肺癌的MDT模式是肿瘤呼吸病学背景下实现肺癌多学科综合治疗的重要模式。本研究团队基于MDT模式^[71]^，提出难治性小细胞肺癌中既往铂类耐药后，肿瘤放射介入科与临床药学结合，立体定向放疗和抗血管生成靶向治疗同时进行的MDT协同。采用治疗前动态影像观察病灶靶区、剂量个体化调整以及用药的副作用的即时监测等措施，经过初步验证，改善了客观缓解率（objective response rate, ORR）以及中位无进展生存期（median progression-free survival, mPFS），显示了跨学科协作治疗的临床应用价值。

充分利用DI-ILD的特殊性，特别是药物性肺纤维化的特点，以及药物性肺纤维化高危患者亚型及异质性的多样性，可以构建“风险评估-动态监测-精准干预-全程管理”为一体的全链条诊疗体系。具体做法包括：（1）风险评估：肿瘤科、呼吸科及遗传学团队协作，利用药物基因组学（如CYP450、HLA-DRB1*04:05等位基因）及影像组学（AI辅助HRCT）预测DI-ILD风险。例如，有GWAS发现MUCSB rs35705950高危基因型和肺纤维化密切相关（OR=5.06）^[48]^，此类患者应提前避免肺毒性较大的药物。（2）动态监测：基于云计算AI系统集成诊疗信息，提前做出监测报告^[72]^。如HRCT提示新磨玻璃影或肺一氧化碳弥散量（diffusing capacity of the lungs for carbon monoxide, DLCO）连续下降时，系统即时推送预警至呼吸科进行损伤评估，从而避免漏诊。临床药师结合抗肺癌药物的药代动力学特性与毒性阈值，联合肿瘤科调整个体化用药剂量。（3）精准干预：呼吸科主导抗纤维化药物（吡非尼酮/尼达尼布）与抗肿瘤治疗的联合方案。例如，卡铂、依托泊苷和尼达尼布的组合可能成为抗肺癌药物所诱发ILD的标准治疗选择之一^[73]^。（4）全程管理：开发集症状自评、用药提醒及影像上传于一体的移动App，实现“医-康-家”三级体系，提升治疗依从性。康复科团队对肺功能、运动耐力（6 min步行距离）和心理（焦虑/抑郁量表）状态的定期检查制定支持方案，提高远期功能预后。

### 5.4 未来展望

抗肺癌药物疗效和安全性的有效管理是未来的研究重点，基于肺癌患者的自我管理与教育，有利于更早发现DI-ILD并将患者纳入自我管理，提高依从性和预防相关合并症（如肺功能的进行性衰退和感染），完善药物审评与监管机制，开展有效的药物不良事件的监测以确保临床用药安全；有利于基因检测技术加速转移和有效实施，推动DI-ILD的管理模式从传统的经验治疗模式向基于生物标志物的精准预防与管理转变；从技术层面推荐整合基因（如GWAS）与药物基因组学信息相结合，结合深度学习驱动的SNP-表型关联分析系统筛查TGF-β等关键信号通路的遗传易感位点，建立中国人群差异的风险评分模型指导剂量的调整及用药的选择。

随着肺癌诊疗技术的迭代、患者健康教育全程化、基因组学研究的深化及跨学科协同机制的完善，抗肺癌药物的治疗获益与药物不良反应有望实现更优平衡，最终为患者延长生存期并改善生命质量。
